# Hemostatic and Tissue Regeneration Performance of Novel Electrospun Chitosan-Based Materials

**DOI:** 10.3390/biomedicines9060588

**Published:** 2021-05-21

**Authors:** Volodymyr Deineka, Oksana Sulaieva, Mykola Pernakov, Viktoriia Korniienko, Yevheniia Husak, Anna Yanovska, Aziza Yusupova, Yuliia Tkachenko, Oksana Kalinkevich, Alena Zlatska, Maksym Pogorielov

**Affiliations:** 1 Medical Institute, Sumy State University, 40007 Sumy, Ukraine; csd@csd.com.ua (O.S.); m.pernakov@med.sumdu.edu.ua (M.P.); v.kornienko@med.sumdu.edu.ua (V.K.); e.gusak@med.sumdu.edu.ua (Y.H.); yanovskaanna@gmail.com (A.Y.); ms.aziza.yusupova@gmail.com (A.Y.); y.tkachenko@med.sumdu.edu.ua (Y.T.); 2 Medical Laboratory CSD, 03148 Kyiv, Ukraine; 3 Institute of Applied Physics, 40000 Sumy, Ukraine; oksana.kalinkevich@gmail.com; 4 Biotechnology Laboratory Ilaya Regeneration, Medical Company Ilaya, 03115 Kyiv, Ukraine; office@ilaya.ua; 5 State Institute of Genetic and Regenerative Medicine of NAMS of Ukraine, 04114 Kyiv, Ukraine; 6 NanoPrime, 39-200 Dębica, Poland

**Keywords:** chitosan, electrospinning, hemostasis, regeneration

## Abstract

The application of chitosan (Ch) as a promising biopolymer with hemostatic properties and high biocompatibility is limited due to its prolonged degradation time, which, in turn, slows the repair process. In the present research, we aimed to develop new technologies to reduce the biodegradation time of Ch-based materials for hemostatic application. This study was undertaken to assess the biocompatibility and hemostatic and tissue-regeneration performance of Ch-PEO-copolymer prepared by electrospinning technique. Chitosan electrospinning membranes (ChEsM) were made from Ch and polyethylene oxide (PEO) powders for rich high-porous material with sufficient hemostatic parameters. The structure, porosity, density, antibacterial properties, in vitro degradation and biocompatibility of ChEsM were evaluated and compared to the conventional Ch sponge (ChSp). In addition, the hemostatic and bioactive performance of both materials were examined in vivo, using the liver-bleeding model in rats. A penetrating punch biopsy of the left liver lobe was performed to simulate bleeding from a non-compressible irregular wound. Appropriately shaped ChSp or ChEsM were applied to tissue lesions. Electrospinning allows us to produce high-porous membranes with relevant ChSp degradation and swelling properties. Both materials demonstrated high biocompatibility and hemostatic effectiveness in vitro. However, the antibacterial properties of ChEsM were not as good when compared to the ChSp. In vivo studies confirmed superior ChEsM biocompatibility and sufficient hemostatic performance, with tight interplay with host cells and tissues. The in vivo model showed a higher biodegradation rate of ChEsM and advanced liver repair.

## 1. Introduction

Liver surgery is a complex procedure following liver laceration, tumors, colorectal cancer metastases or liver transplants. However, bleeding is a common occurrence causing complications during such operations. Hemostasis can be done in various ways, such as mono or bipolar electrocautery, sealing devices like Ligasure^®^ (Minneapolis, MN, USA), argon-plasma coagulation, clipping of available tubular structures, suturing, application of topical hemostatic materials and wound tamponade [1]. Among these various strategies, application of topical hemostatic materials (THMs) showed high efficacy. THMs facilitate coagulation and prevent bleeding recurrence, thus reducing operating times. Furthermore, it also decreases the amount of blood needed for blood transfusion, and re-laparotomy probability [2].

A majority of THMs are of a biological origin and act as active and passive agents. Active THMs made from blood components (e.g., thrombin or fibrin) directly affect coagulation, leading to rapid blood clotting. Passive THM, made from cellulose, gelatin, collagen, etc., act by absorbing plasma and aggregating blood cells, leading to the formation of a matrix for better clotting [3]. The implementation of active THMs in surgical practices has significantly improved clinical outcomes. However, their application is limited due to their high cost, contamination risks, short shelf life, low portability, performance variability and immunological side effects [4]. Despite the wide range of THMs available in the medical market, there is still exists a need to develop new materials. High hemostatic performance, safety, low cost, simple preparation, excellent biodegradability and biocompatibility are the desired attributes of such a material or materials [5].

In this regard, chitosan (Ch), a natural polycationic polysaccharide mainly obtained from chitinous shell crab or certain fungi, is a promising biopolymer demonstrating both hemostatic properties and high biocompatibility. It shows high biocompatibility, biodegradability, nontoxicity and bacteriostatic properties. The hemostatic capability of chitosan is manifested by aggregation of red blood cells (RBCs), activation of platelets (PLT) and contact activation system [6,7]. The amino and hydroxyl groups of Ch are similar to glycosaminoglycans of the liver, which makes it most suitable to create hepatic extracellular matrix [8]. Properties of chitosan for such applications strongly depend on the molecular weight, the degree of deacetylation and the physical form of the material [9]. The chitosan is most effective to cause blood aggregation with 75–88% of deacetylation and 50–190 kDa molecular weight [10]. Furthermore, chitosan can be used in various forms, such as a gel, film, sponge, aerogel or membrane. For hemostatic purposes, it is preferable to use sponges or powders based on chitosan, which has significant sorption and is easy to use [11]. The critical limitation for Ch-based sponge is its biodegradation time that can lead to long-term postoperative complications and decrease liver regenerative potential. These shortcomings desire (or demand) new technologies to improve biocompatibility and biodegradation of Ch-based materials. Electrospinning techniques is one such option which provides the possibility of nanofibrous structure formation with low density that can overcome these limitations [12].

Electrospinning allows us to build three-dimensional porous, fibrous materials, using synthetic and natural polymers such as chitosan [13]. This method is used together with 3D printing in biomedicine and tissue engineering [14]. Electrospinning scaffold models the extracellular matrix for rapid cell proliferation and tissue regeneration [15]. Previous studies showed that electrospun chitosan membranes have high blood clot-forming activity and biocompatibility. However, the formation of hydrogen bonds after the dissolution of chitosan in an acid solution causes great difficulties in the electrospinning process, which severely limits the use of pure chitosan [16]. To improve the spinning of chitosan, reduce electrical conductivity and obtain thinner fibers, additional polymers, such as caprolactone (PCL), polyvinyl alcohol (PVA) or polyethylene oxide (PEO), are used [17]. We assumed that the best solution to stabilize the chitosan electrospinning process would be the addition of PEO as a water-soluble synthetic polymer with good biocompatibility and low toxicity [18]. In this study, we used PEO, which binds to chitosan due to hydrogen bonds, reducing the chitosan solution’s viscosity, surface tension and conductivity, which makes it possible to obtain a dense network of nanofibers [19,20]. However, there are limited data on the hemostatic properties of Ch-PEO copolymers [21]. This study was designed to assess the hemostatic performance, biocompatibility and degradation of newly produced Ch-PEO-copolymer prepared by electrospinning compared to Ch-sponge.

## 2. Materials and Methods

### 2.1. Fabrication of Chitosan Hemostatic Materials

Two types of chitosan-based hemostatic materials were prepared as follows:

(1) Chitosan hemostatic sponge (ChSp) was prepared using 300 kDa molecular weight chitosan (Ch) with 95% deacetylation degree and 1% acetic acid purchased from YuDa Chemicals, Qingdao, PRC. Chitosan was dissolved in acetic acid and stirred for 24 h at room temperature until it becomes homogeneous. This solution was then placed in a reaction vessel with a maximum column height of 1.0 cm. The polymer solution was frozen at −25 °C for 24 h and then dried in the vacuum chamber (0.1 Pa, 24 h).
(2) Chitosan electrospinning membranes (ChEsM) were prepared from chitosan powder, 300 kDa molecular weight (95% deacetylation degree) and polyethylene oxide (PEO) powder (400 g/mol) purchased from Glentham Life Sciences (Corsham, UK). Ch (2 g) and PEO (3 g) were dissolved in 100 mL of 50% acetic acid and stirred for 24 h at room temperature. After complete dissolution, solutions were mixed in a 3:1 PEO/Ch ratio. The conductivity of this solution was 1487 μS/cm. The electrospinning process was performed at room temperature and relative humidity of 15–20% in the safe cabinet with laminar airflow in the RT-Advanced machine (Linari Engineering, Pisa, Italy). Ch/PEO solution was poured into a 10 mL glass syringe with a needle diameter of 0.6 mm. The distance between the needle and the collector was 12 cm. The parameters of electrospinning were set as follows: the flow rates, 0.2 mL/h; the voltage applied to the needle, 17 kV; the rotational speed of the collector (10 cm in diameter), 800 rpm. The electrospun membrane was vacuum dried at room temperature within 12 h.

### 2.2. Scanning Electron Microscopy (SEM)

The samples were sputter-coated with a thin layer of silver (30–50 nm) under vacuum setup VUP-5M (SELMI, Sumy, Ukraine) and visualized with SEO-SEM Inspect S50-B (FEI, Brno, Czech Republic) scanning electron microscope. Assessment of nanofiber morphology and mesh pore analysis were based on SEM images, using DiameterJ plugin 1.018w for Fiji (distribution ImageJ 1.51w, University of Wisconsin, Madison, WI, USA).

### 2.3. Porosity and Density

The porosity (p, %) and density (d, g/cm^3^) of the chitosan scaffold were measured by isopropanol displacement. Each sample of equal weight (W, g) was placed in the pre-determined volume (V, cm^3^) of isopropanol. The volume change of the alcohol-impregnated samples was measured after a fixed time (5 min). Then hemostatic material was removed from the isopropanol, and the difference in isopropanol volume was measured. The porosity (Equation (1)) and density (Equation (2)) were calculated using the following equations:

p = (V_1_ − V_3_)/ (V_2_ − V_3_) 100% (1)

d = W/ (V_2_ − V_3_) (2)

where d is density, g/cm^3^; p is porosity, %; W is the weight of the sample, g; V_1_ is the initial volume of isopropanol, cm^3^; V_2_ is the volume of isopropanol with the immersed sample, cm^3^; and V_3_ is the volume of isopropanol after sample removal, cm^3^.

All experiments were repeated three times.

### 2.4. Degradation and Biodegradation Study

Degradation (D) studies of chitosan hemostatic materials were performed in simulated body fluid (SBF). SBF was prepared by using NaOH, NaCl, NaHCO_3_, KCl, KH_2_PO_4_ ·3H_2_O, MgCl_2_·6H_2_O, CaCl_2_ and Na_2_SO_4_ purchased from POCH, Gliwice, Poland. SBF had a pH 7.4 and ion concentrations approximately equivalently to that of human blood plasma. SBF solution was sterilized by filtration, using a 0.2 μm filter, and biomaterials were sterilized by using an autoclave. All degradation tests were performed for seven days. The pre-weighed chitosan samples were immersed in 50 mL of sterile SBF solution. The hemostatic materials were removed and washed with distilled water, followed by drying and weighing every 24 h.

Biodegradation (B) studies were conducted in vitro, using human lysozyme, which is a glycosidic hydrolase enzyme hydrolyzing β-glycosidic bonds. Lysozyme is commonly found in human secretions such as a tears, saliva and mucus at the concentration of 7–13 mg/L. For this study, pre-weighed samples (W_0_, g) were immersed in sterile SBF containing lysozyme (10 mg/L) at 37 °C to simulate environment in human body. The chitosan materials were taken out, washed with distilled water, dried and weighed (W_t_, g) every 24 h. All experiments were repeated three times. Degradation and biodegradation were calculated by using the following equation:

(B)D = (W_0_ − W_t_)/W_0_ 100% (3)

### 2.5. Antibacterial Test

Antibacterial properties of the scaffolds were investigated against a Gram-negative bacterium, *Escherichia coli* (*E. coli*, B 926) and a Gram-positive *Staphylococcus aureus* (*S. aureus*, B 918) obtained from the Bacteria Collection of Sumy State University. All bacteriological media were purchase from HiMedia (Maharashtra, India). The bacterial cultures were incubated in nutrient broth for 24 h. Bacterial suspension was then diluted to achieve a final bacterial concentration (or count) of 106 colony forming units (CFU)/mL for inoculation. Each sample was placed in the tubes with 2 mL of bacterial inoculum and incubated for 2, 4, 6, 8, 10 and 24 h at 37 °C. The samples tested in the nutrient medium (no bacteria) served as a negative control. The bacterial suspension containing 6 log10 CFU/mL was a positive control. At the specified time intervals, 100 μL aliquots from tubes were transferred onto the agar plates’ and incubated at 37 °C for 24 h to determine the bacterial survival. The test was done in triplicate.

### 2.6. Cell Viability Assay

Alamar Blue colorimetric assay and Live/Dead staining were performed to evaluate the effect of chitosan hemostatic materials on the viability of U2OS cell lines. U2OS was obtained from Umeå University (Umeå, Sweden). The cells were cultured in Dulbecco’s Modified Eagle’s Medium/Nutrient Mixture F-12 with L-glutamine contained 100 units/mL penicillin, 100 μg/mL streptomycin, 2.5 μg/mL amphotericin B, 10% fetal bovine serum (FBS) and 1.0 ng/mL bFGF (basic Fibroblast Growth Factor) at 37 °C in a humidified atmosphere with 5% CO_2_. All media and reagents were purchased from Gibco^®^ and Invitrogen^®^, Thermo Fisher Scientific, Gaithersburg, MD, USA. The medium was changed every other day until the cells became confluent. For the Alamar Blue test, the cells were seeded onto 24-well plates at a density of 2 × 10^4^ cells/well. After overnight incubation, the weighed chitosan strip (40 mg) was added in each well. After 24 h, Alamar blue was added in an amount equal to 10% of the volume to each well. The wells containing only cells without samples accounted for as a control. The plates were incubated for four h at 37 °C in the dark. A total of 100 μL of medium from each well was transferred to another 96-well plate, and the absorbance was measured by using a Multiskan FC (Thermo Fisher Scientific, Waltham, MA, USA) plate reader at wavelengths of 570 and 600 nm. The percentage reduction of Alamar Blue was measured at different time intervals: 1st, 3rd and 7th day. All experiments were repeated three times.

### 2.7. Live/Dead Staining

The experiments with the use of human cell culture in vitro were carried out following the human experiment issues of the Code of Ethics of the World Medical Association (Declaration of Helsinki, 19 October 2013). In all cases, donors of ADSCs were the informed voluntary. Adipose-derived Stem Cells (ADSCs) were isolated from the lipoaspirate by enzymatic digestion in 0.1% collagenase IA and 0.1% pronase with 2% fetal bovine serum (FBS). This cell suspension was then transferred to 25 cm^2^ cell culture flask (SPL, Gyeonggi-do, Korea) and cultured in the following control growth medium: modified MEM-α with 10% FBS, 2.0 mM L-glutamine, 100 U/mL penicillin, 100 μg/mL streptomycin and 1.0 ng/mL bFGF-2. (All media and reagents were obtained from Sigma-Aldrich, St. Louis, MO, USA.) The cells were cultured in multi-gas incubator CB210 (Binder, Tuttlingen, Germany) at +37 °C under saturated humidity, 5% CO_2_ and 5% O_2_. For the cytotoxicity assessment, the cells were seeded on scaffolds at a density of 2 × 10^5^ cells per 1.0 cm^3^ of materials. After 48 h of incubation, samples were stained with PI (propidium iodide) and FDA (fluorescein diacetate). The number of dead and living ADSCs in different groups was counted, using fluorescence microscopy (FITC and Texas Red filters; Carl Zeiss, Jena, Germany) and ZEN 2012 software [22].

### 2.8. Blood Interaction Test

Whole blood (40 mL) was obtained from 2 human volunteers by a registered nurse at the Medical Institute of Sumy State University. The study was previously approved by the Ethics Committee on Medical Research of Medical Institute Sumy State University (protocol 10/7 from 15 October 2019). Appropriate informed consent was obtained from all volunteers. Human whole blood was immediately placed to Becton Dickinson Vacutainers^®^ (Mississauga, ON, Canada) with 3.6 mg EDTA of 2 mL in which the strips of chitosan hemostatic material weighted 40 mg (W_1_, mg) were previously placed. Over the next 10 min, vacutainers were shaken to provide the interaction between the sample and blood. All materials were removed from Becton Dickinson Vacutainers^®^ (Mississauga, ON, Canada) and weighted (W_2_, mg). Blood sorption (BS) rate was calculated as follows:

BS = W_2_ − W_1_ (4)

The remaining blood was used for a completed blood count (CBC) test for the study of adhesion and aggregation of blood cells. The CBC test was performed on the hematology analyzer CELL-DYN 3700 (ABBOTT, Irving, TX, USA), using reagents DIAGON (Budapest, Hungary). The following parameters were evaluated: platelet count (PLT, ×10^9^ /L), platelet distribution width (PDW, %) and mean platelet volume (MPV, fL). After weighing, the samples are placed in 2% glutaraldehyde for fixation for 2 h, then transferred to ethanol of ascending concentration for stepwise dehydration. All samples after drying were covered with a 30–50 nm layer of silver for SEM.

### 2.9. Animals and Surgical Procedures

The experimental protocol was reviewed and approved by the Ethical Committee of Sumy State University (protocol number: 5/2; date of approval: 12 May 2020). The Guide for the Care and Use of Laboratory Animal (1996) and Directive 2010/63/EU of the European Parliament and the Council on the Protection of Animals Used for Scientific Purposes (2010) were followed. 

Thirty 24-week-old male white laboratory rats (Wistar line) with a body weight of 250–300 g were housed in a temperature-controlled room (25 °C) under a 12/12 h reversed day/night cycle and received food and water ad libitum. All rats were randomly assigned to two groups (*n* = 15/group) to evaluate the hemostatic effect of chitosan materials: I–ChSp and II–ChEsM.

**Figure 1:**
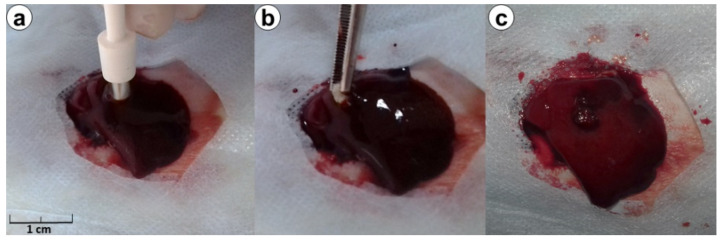
Procedure of punch biopsy liver trauma (**a**), hemostatic application (**b**) and liver after hemostasis procedure (**c**).

Trauma-hemorrhage model of rat’s liver: The animals were anesthetized by intraperitoneal injection of 7 mg/kg ketamine (Farmak JSC, Kyiv, Ukraine) and 10 mg/kg xylazine (Alfasan International B.V, Woerden, Netherlands). The rats were restrained in a supine position and shaved, and a 2 cm midline laparotomy was made under sterile conditions. The left medial lobe of the liver was brought out through the incision. A penetrating 4-mm punch biopsy was performed (Figure 1) from the diaphragmatic surface on full-thickness. It was to simulate bleeding from a non-compressible irregular wound. Immediately after, the liver trauma started to applying hemostatic material according to the group. Duration of bleeding was assessed from the start of the application until hemostasis was obtained based on time measurements. After the bleeding stopped, the abdomen wound was closed with two-layer continuous 4/0 Vicryl (Ethicon Inc., Johnson & Johnson Co., Arlington, TX, USA) sutures.

This section may be divided by subheadings. It should provide a concise and precise description of the experimental results, their interpretation and the experimental conclusions that can be drawn.

### 2.10. Histological Tissues Processing

The animals were sacrificed after 1, 4 and 8 weeks after surgery by narcosis overdose ketamine (100 mg/kg). The liver tissues were taken for histology investigation and fixed in 10% neutral buffered formalin for 24 h with further processing according to the standard protocols. Paraffin-embedded blocks were cut at 4 μm thickness and stained by hematoxylin and eosin. Cross-sections of lesions with the implanted hemostatic agents were analyzed histologically. The primary histopathological assessment was performed microscopically. In addition, digital images of sections were captured using a digital slide scanner (3DHISTECH, Budapest, Hungary) and assessed by two independent observers. The histological evaluation included an assessment of the following aspects: the presence and expansion of inflammation and tissues ingrowth inside the hemostatic materials, the pace of biomaterials degradation, the interaction between biomaterials and host cells/tissues. To assess the pace of hemostatic biomaterials degradation their diameters were measured. The thickness of the capsule demarcating hemostatic agents from the liver was estimated to examine the host tissues reaction against the implanted biomaterials. The evaluation of inflammatory infiltration and tissue ingrowth was conducted using the semi-quantitative score system (from 0 to 3: 0—absent, 1—mild, 2—moderate and 3—severe/significant). For this aim, the observers scored every sample in 5 spots within the hemostatic material and around it at high magnification. Finally, the size of liver defects was measured during every term to evaluate the repair of the liver lesion.

### 2.11. Immunohistochemical Study

Sequential sections 4.0 μm in thickness were used for immunohistochemical analysis. The liver tissues were de-paraffinized and hydrated, and the endogenous peroxidase activity in them was blocked by using 3% methanol in hydrogen peroxide. Next, antigen retrieval in a water bath at 98 °C was performed by using TRIS EDTA or citrate buffer (pH6) and followed by incubation with primary antibodies. After washing, labeled polymer secondary antibody (Envision Detection System, Dako) was added to the slides, and peroxidase activity was detected by using diaminobenzidine (DAB)—tetrahydrochloride liquid plus Chromogen System (Dako) substrate. The reaction was stopped with distilled water, and the sections were counterstained with hematoxylin and mounted in Richard-Allan Scientific Mounting Medium (ThermoFisher, Waltham, MS, USA). CD68 (DAKO, Clone KP1) and CD163 (Cell Marque, Clone MRQ-26) were used to visualize macrophages of different phenotypes (M1 and M2 respectively). The cytotoxic T cells were identified by CD8 (DAKO; Clone C8/144B). FOXP3 (Cell Marque, Clone EP340) was used for the investigation of T-regulatory lymphocytes producing anti-inflammatory and profibrogenic agents. The number of immunopositive cell was counted in the capsule demarcating biomaterial from the surrounding liver tissues with further recalculation per 1 mm^2^.

### 2.12. Statistical Analysis

Quantitative data were expressed as mean ± standard deviation or mean and interquartile range (IQR). Comparisons between groups were performed, using the *t*-test. In addition, Kruskal–Wallis test was used when working with categorical data. *p* < 0.05 were figured statistically significant. Statistical analyses were performed with GraphPad Prism 8.0 (GraphPad, San Diego, CA, USA).

## 3. Results and Discussion

### 3.1. SEM

**Figure 2:**
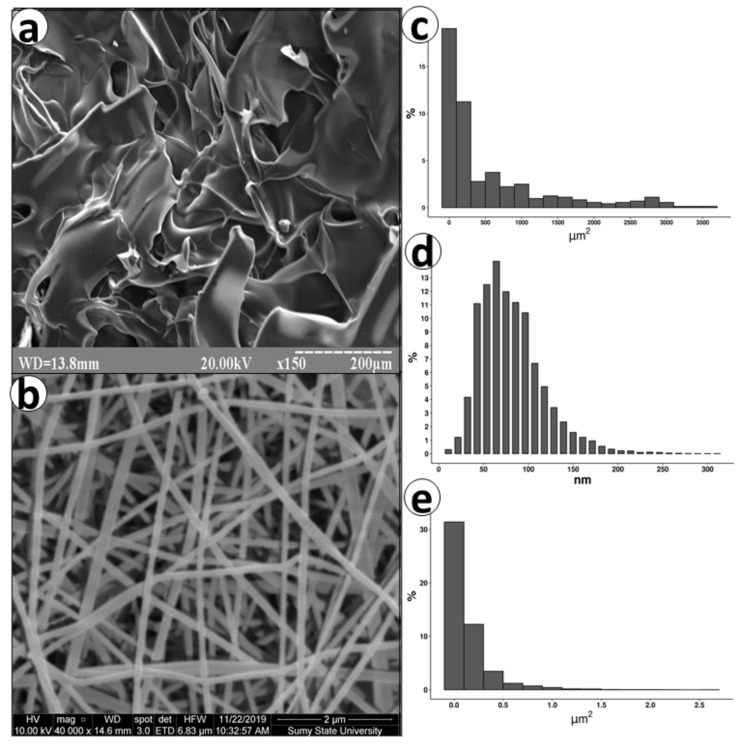
Scanning electron microscopy image of ChSp (**a**) with analysis of pore area (**c**). Structures (**b**), fiber diameter (**e**) and pore area (**d**) of ChEsM.

The Ch-based materials exhibited adequate porosity with different structures depending on the manufacturing method (Figure 2). ChSp was made of differently shaped flakes that surround pores with a median of pore area 164 µm^2^ (IQR 47 to 767). The large open pores could accelerate the absorption of liquid that is essential for hemostatic materials. The plates of the ChSp had a tortuous surface, which additionally increased the effective contact area. Opposite to ChSp, ChEsM structured from the randomly oriented fibers with the median thickness of 160 nm (IQR 86 to 236). Some research demonstrated that randomly oriented fibers exhibit advanced tissue regeneration potential due to similarity with the extracellular matrix [23,24]. It is considered that a nanometer fiber diameter promotes faster biodegradation of the ChEsM [25]. In contrast to ChSp, the median pore area of ChEsM was 0.06 µm^2^ (IQR 0.02 to 0.16), The homogeneity of fibers and pores might predict the equal interaction with blood cells with increased hemostatic ability.

### 3.2. Porosity and Density

**Figure 3:**
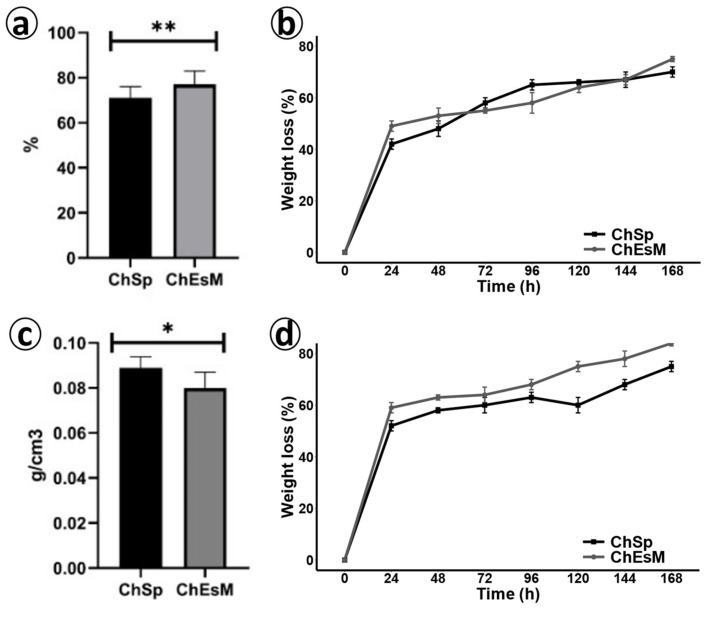
The porosity (**a**), density (**c**), degradation in SBF(**b**) and biodegradation in lysozyme solution (**d**) of hemostatic materials; * (*p* ≤ 0.05) and ** (*p* ≤ 0.001)—the significant between two groups.

The measurements of isopropanol displacement (Figure 3a,c) showed that ChEsM had a significant higher porosity of 77 ± 3% vs. ChSp 71 ± 5% (*p* < 0.001). The lower porosity of the sponges is probably due to the large number of closed pores. The density of chitosan-based materials was significantly lower (*p* < 0.05) for the ChEsM 0.08 ± 0.07 g/cm^3^ whereas for ChSp it was 0.089 ± 0.05 g/cm^3^. The mass of the material used to create a unit volume of hemostatic material primarily depends on the method of synthesis, as well as the properties of the polymer. The density of the topical hemostatic adds a skeletal function that is necessary to tamponade the wound [26]. However, after the bleeding stops, the tightly packed sponge will be slower to resorb [27].

### 3.3. Degradation 

Degradation research showed that both materials rapidly lost weight in the SBF and lysozyme solution (Figure 3b,d). During the first 2 days of the study, the degradation rate was higher for the ChEsM and reached 53%. On days 3 and 4, ChEsM degradation became slower; however, it was significantly (*p* < 0.05) greater than those of ChSp (65% vs. 58%). In the next two days, the rate of degradation of the ChSp stabilized, and at the end of the study (day 7), it reached 70%. ChEsM degradation, on the other hand, gradually accelerated and reached 75%, which was significantly higher (*p* < 0.05) compared to the ChSp. 

Biodegradation in lysozyme solution was slightly different from degradation in FBS solution. Notably, on the first day of the study, weight loss in lysozyme was 10% higher for both samples comparing to FBS solution. Enzymatic cleavage of the ChSp was more uniform, and on day 7, it reached 75% weight loss. In contrast, there was acceleration of the ChEsM biodegradation rate from day 5 (*p* < 0.05) that resulted in 84% weight loss at the end of the study. For topical hemostatic materials, a balance of efficiency and the rate of degradation is essential [28]. A too-rapid degradation increases the risk of re-bleeding, and a too-long degradation can disrupt organ regeneration [29].

### 3.4. Antibacterial Properties

**Figure 4:**
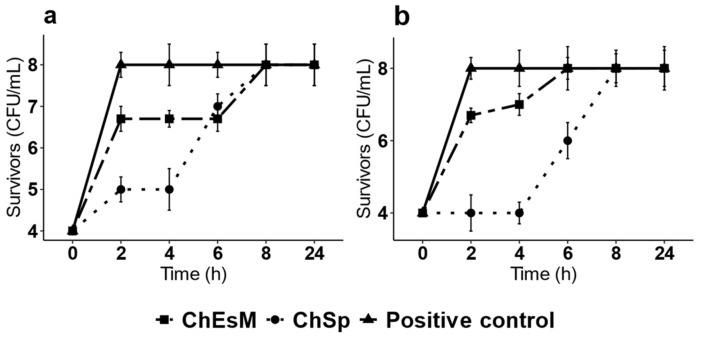
Effect of ChSp and ChEsM on the survival of *S. aureus* (**a**) and *E. coli* (**b**).

There is a common risk of wound infection during bleeding due to the absence of protective barriers in the body. The hemostatic materials, such as sponges must possess antibacterial properties to decrease the risk of systemic infection [30]. Two representative bacteria *(S. aureus*, a Gm+, and *E. coli*, a Gm− bacteria) were used to analyze the antibacterial activity of sponges and ChEsM membranes. The growth curves of both strains after incubation with ChEsM and ChSp during 24 h are shown in Figure 4. Moreover, as shown the positive control, the untreated *E. coli* and *S. aureus*, showed typical growth phases. Studies showed that both test organisms grew actively within 2 h in ChEsM treated samples. Furthermore, the bacterial population was the same as compared to the positive control at the end of 6 h for *E. coli* and 8 h for *S. aureus*. Even at the end of 24-h study duration, results were no different. Therefore, it suggests that ChEsM does not show good antibacterial activity against both bacteria. It indicates that modification of chitosan by electrospinning did not improve its antibacterial activity. The ChSp showed slightly better antimicrobial activity compared to ChEsM, and it was more pronounced against Gram-negative bacteria. The ChSp was effective in its antimicrobial activity against both strains for 6 h. However, after 8 h of co-cultivation with these samples, the bacterial population (the CFU/mL) was the same compared to the positive control data. It may be due to its lower degradation rate and thus prolonged inhibitory effect of ChSp in contrast with ChEsM [31]. Therefore, the ChSp sponge can be more effective to prevent bacterial growth during first hours after surgery.

### 3.5. Cell Viability Assay

**Figure 5:**
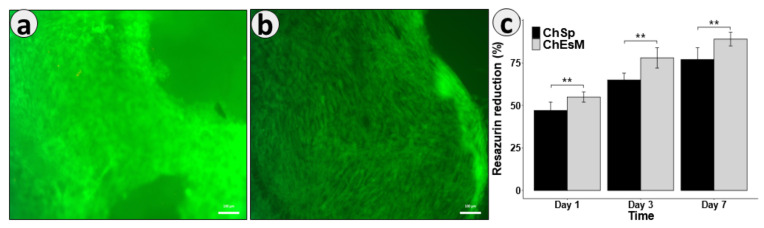
ChSp (**a**) and ChEsM (**b**) live/dead staining with FDA/PI after 48 h of cell cultivation and cell viability ChSp and ChEsM, using Resazurin reduction assay (**c**); **—the significant difference (*p* ≤ 0.001) between two groups at the same timepoint.

Both hemostatic materials showed their non-toxicity after 48 h of cultivation and live/dead staining with FDA/PI (Figure 5a,b). The cells were well attached and evenly distributed on the surface of the samples. 

Resazurin reduction assay (Figure 5c) demonstrated significantly (*p* < 0.05) better cell adhesion on the first day for ChEsM. Adhesion properties are necessary both for fast hemostasis and for acceleration of start of regeneration processes. The ChEsM had significantly (*p* < 0.05) higher cell proliferation on its surface on both day 3 and day 7 of cultivation. The structure of the membrane facilitated the growth of cells on its surface and the penetration of cells into deeper layers. This is important for complete biodegradation of hemostatic material and repair of the damaged organ.

### 3.6. Blood Interaction Test

**Figure 6:**
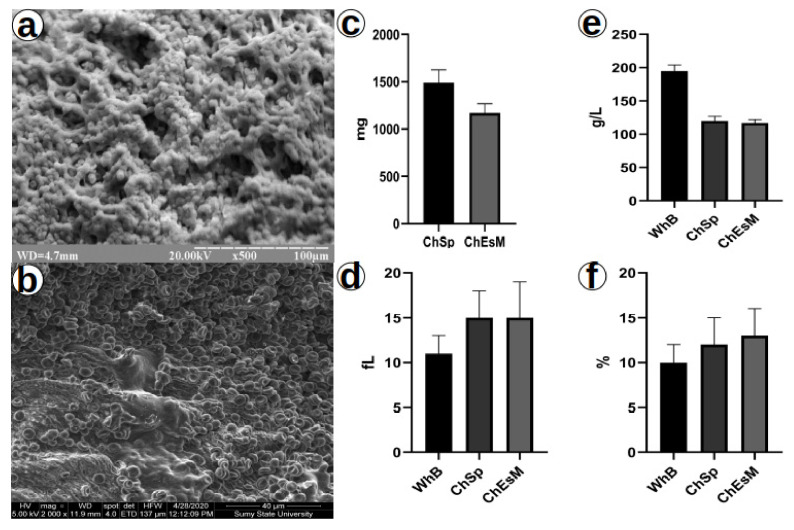
SEM of ChSp (**a**), ChEsM (**b**), blood sorption (**c**) and hematological parameters: platelet (**e**), platelet distribution width (**d**) and mean platelet volume (**f**) after interacting with blood.

After interaction with whole blood (WhB), both materials adhered to a significant number of blood cells on their surface (Figure 6). The ChSp showed significantly (*p* < 0.05) higher blood sorption compared to the ChEsM. Both hemostatic materials significantly (*p* < 0.05) reduced platelet counts with no difference between groups. Chitosan hemostatics showed no differences (*p* > 0.05) for MPV and PDW; however, there was a significant (*p* < 0.05) difference with control. These hematological parameters may indicate platelet activation, which is an essential component of the first phase of hemostasis [32].

### 3.7. In Vivo Results

In the hemostatic study, no animal died. The animals from all experimental groups did not differ significantly in weight age, blood loss before the study or weight of the injured liver lobe. No hemodynamic disorders or worsening of behavioral responses were found in rats. No evident diversity in the bleeding time was observed (*p* > 0.05). ChSp completely stopped the bleeding for 80.6 ± 5.7 s, and for the ChEsM time, full hemostasis was 84.5 ± 4.8 s. A second application of a hemostatic agents was not necessary in any case.

### 3.8. Histological and Immunohistochemical Study

One week after surgery and biomaterials application, the histologic analysis found that both hemostatic agents adapted well to the liver defect. THMs filled in the cavities made during punch biopsy and were separated from liver tissues by a thick capsule made by immature connective tissue rich numerous cells. Importantly, the liver injury and THM application were accompanied by the inflammatory reaction that differed in intensity and cellular composition depending on the applied biomaterial. Additionally, the features of active regeneration were found at the boundary with surrounding liver tissues one week after surgery.

**Figure 7:**
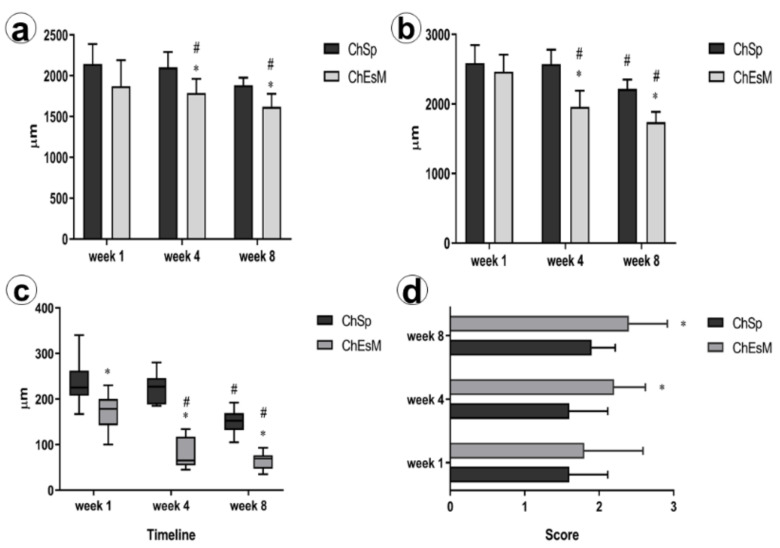
Dynamic changes of liver lesion size and biomaterial degradation: (**a**) shows the dynamic of hemostatic plugs and (**b**) reflects the changes in liver defect size over the time of experiment; (**c**) thickness of the capsule surrounding hemostatic materials; (**d**) host tissues ingrowth within hemostatic plug. * Shows significant differences between groups at *p* < 0,05; # demonstrated differences when compare with previous term.

When assessing the dynamics of in vivo biodegradation of THMs, we found that despite the similar size of implanted materials at the early terms after surgery (*p* = 0.123 in 1 week), the diameter of ChEsM declined progressively by 4th (*p* = 0.005) and 8th weeks (*p* = 0.0001) compared to ChSp. As a result, at the end of the experiment, the ChEsM diameter was about twice smaller than its initial size (Figure 7a,b). In contrast, the pace of ChSp biodegradation was much slower.

Correspondingly, the size of the liver displayed similar characteristics. While ChSp remained unchanged during the 1st month after surgery, rats with ChEsM application demonstrated a gradual reduction of the liver tissue defect. This was associated with thinning of the capsule around the ChEsM (Figure 7c) and progressive tissues ingrowth within THM meshwork (*p* = 0.014 in week 4 and *p* = 0.002 at week 8). Less explicit was the contraction of the liver defect in rats with ChSp application that correlated with tissues ingrowth within hemostatic sponge. It worth noting, that varieties in biomaterials structure affected the interplay with host cells. In addition to connective tissue ingrowth (Figure 7d) and numerous giant foreign bodies macrophages in both groups, ChEsM made a basis for cell adhesion. Since the 1st week after surgery, there were features of cell layers lining on the electrospun membranes.

**Figure 8:**
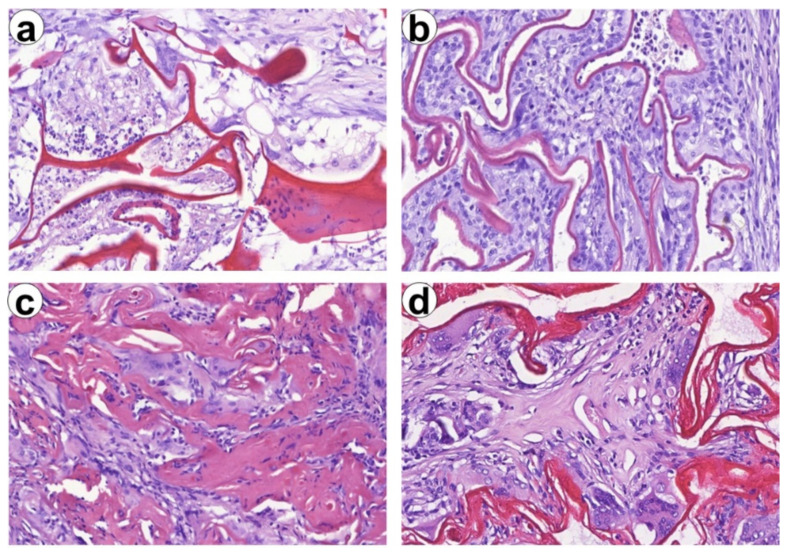
Differences in Ch-based materials degradation and biocompatibility at different terms after surgery. (**a**,**b**) Hemostatic materials (ChSp and ChEsM, respectively) at early stages after surgery (after one week). (**c**,**d**) Same materials in 2 months after liver trauma, Magnification 400×.

The differences in material thickness and interplay with host cells and tissues were distinguishable. While ChSp had irregular thickness, ChEsM were arranged in thin-membranes regular pattern (Figure 8). In 2 months after liver resection, slow degradation of ChSp was associated with material aggregation surrounded by giant cells of foreign bodies. ChEsM membranes interfered with host tissues which were ingrown within the remnants of biomaterial.

**Figure 9:**
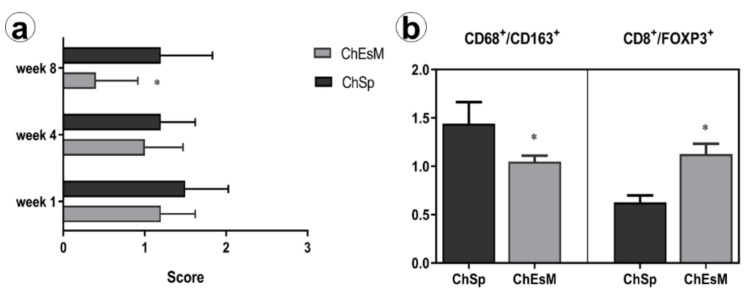
Inflammatory reaction and immune cells ratios in the capsule surrounding hemostatic agents. (**a**) Score of inflammatory infiltration in the capsule surrounding hemostatic materials and separating them from liver tissues. (**b**) Differences in M1/M2-macrophages and CD8/FOXP3-lymphocytes subtypes ratio in the capsule around ChSp and ChEsM; *—significant difference (*p* ≤ 0.05) between two groups.

Inflammation in the capsule was associated with M1 and M2 macrophages recruitment. M2-type macrophages were numerous in perivascular areas and at the outer layer of the capsule (Figure 9b). Moreover, CD8 and FOXP3 cells were found within the capsule. We did not find significant differences in macrophages and lymphocytes subtypes number. However, the CD68/CD163 ratio was shifted towards M2 macrophages in the ChEsM group, while, in ChSp ras, slight Treg-switch was elicited. These findings can reflect differences in immune cell reactions to differently modified chitosan-based materials.

Despite the lack of significant differences in inflammatory reaction score (Figure 9a) at early terms after surgery, in the 8th week of the experiment, the ChEsM group demonstrated lower inflammation and better liver regeneration. Although the absolute number of macrophages and lymphocytes did not demonstrate distinctions between groups, there were some peculiarities between M1/M2-macrophages and CD8/FOXP3 lymphocytes subtypes balance.

**Figure 10:**
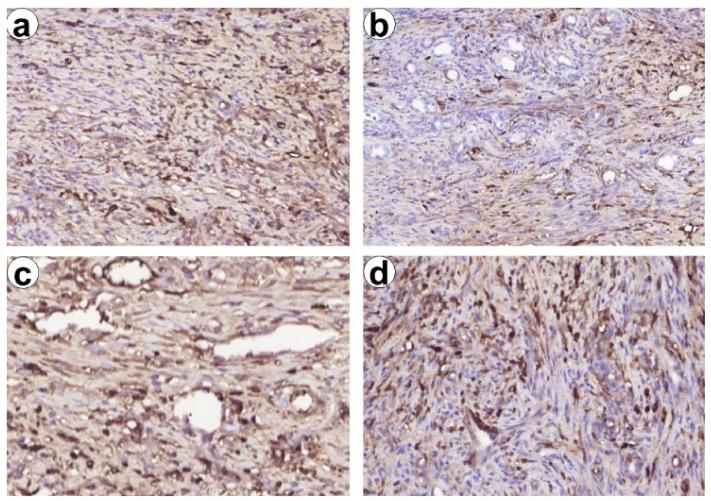
Infiltration of the capsule surrounding chitosan-based materials with CD68+ and CD163+ macrophages (**a**,**b**) and lymphocytes (**c**,**d**) in 7 days after hemostasis. Immunohistochemical study. (**a**) Numerous diffusely spread CD68+ cells in the capsule surrounding liver defect filled with hemostatic material. (**b**) CD163+ cells concentrating predominantly around vessels and cholangioles reflecting their association with repair. (**c**,**d**) CD8+ and FOXP3+ lymphocytes in the capsule surrounding liver defect filled with hemostatic material, Magnification 200×.

Exploring potential mechanisms responsible for improved bioactive properties of ChEsM we compared the number of the immune cells (M1/M2 macrophages and CD8 vs FOXP3 lymphocytes) in the capsule that demarcated material from the liver and reflects host reaction against the material (Figure 10). There are contradictory data about the impact of chitosan and its derivatives on macrophages activation and polarization. Mori T. et al. [33] showed that chitosan activates macrophages, however, it can induce their apoptosis in response to phagocytosis. On the other hand, the prevalence of M2-macrophages polarization after Ch-based materials application was defined, though the role of different degrees of acetylation in macrophage polarization was demonstrated [34].

In our study, we found both M1 and M2 macrophages reaction in ChSp and ChEsM groups. Despite the lack of significant differences in that absolute number, there was a shift towards M2 macrophages in ChEsM group. M2-macrophages are known as anti-inflammatory phenotypes that produce a broad range of growth factors and play a vital role in the resolution of inflammation, angiogenesis and tissue repair [35]. Notably, CD163+ cells were preferably located around the sites of hepatocellular regeneration and angiogenesis that could explain advanced tissues ingrowth and lesion repair in the ChEsM group.

Furthermore, the presence of PEO in ChEsM can affect adaptive immune cells reaction, inducing lymphocytes apoptosis and differentially modulating various lymphocytes activity [36]. Herein we found the shift of CD8/FOXP3 ratio towards T-reg lymphocytes in the capsule of ChSp group that could be a compensatory mechanism to inhibit inflammatory reaction against ChSp as they produce numerous anti-inflammatory mediators important for tissues repair [37]. Moreover, FOXP3 cells are known to be responsible for immune tolerance and ban autoimmunity [38,39].

On the other hand, an increased number of Treg cells can be associated with fibrotic changes [40]. However, to discover the immune contexture of Ch-based materials application and material-immunity interplay further investigations are needed.

The comparison of the histological changes in ChSp and ChEsM groups reveled that nanoscale modification of chitosan can significantly improve its bioactive properties and host tissue regeneration. ChEsM had lower immunogenicity and higher biocompatibility representing a cell-friendly substrate facilitating host cells’ adhesion and advanced tissues ingrowth. Accelerated ChEsM biodegradation was associated with refined liver lesion repair.

## 4. Conclusions

In our current studies, we investigated highly porous nanofibrous Ch-PEO mats made by electrospinning technique (ChESM) in comparison with conventional Ch sponge (ChSp). ChESMs had randomly oriented fibers with a median thickness of 160 nm (IQR 86 to 236) and a median pore area 0.06 µm2 (IQR 0.02 to 0.16). Additionally, ChEsM has a significant higher porosity of 77 ± 3% vs. ChSp 71 ± 5% (*p* < 0.001) but the same degradation rate. New ChEsM demonstrate high biocompatibility and sufficient in vitro hemostatic blood interaction. The in vivo liver-bleeding experiment proved the high hemostatic performance of ChESM material (full time of hemostasis was 84.5 ± 4.8 s) and advanced biodegradation in the late postoperative period, compared to the ShSp. ShEsM demonstrated a lower inflammatory reaction both in early and late postoperative periods and had lower immunogenicity with appropriate host-tissue response. The findings of the study have important implications to develop future effective and cheap multi-dimensional chitosan-based nanofibers for parenchymal bleeding application.

## Data Availability

The data presented in this study are available on request from the corresponding authors.
